# Effectiveness of a technical support program with women’s self-help groups in catalyzing health and nutrition behaviors in Bihar—a multicomponent analysis

**DOI:** 10.3389/fpubh.2024.1389706

**Published:** 2025-01-15

**Authors:** Sudipta Mondal, Indu Bisht, Santosh Akhauri, Indrajit Chaudhuri, Narottam Pradhan, Sweta Kumari, Shuchi Sree Akhouri, Rakesh Kumar Jha, Manoj Kumar Singh, Suman Das, Apollo Purty, Arko Mukherjee, Tanmay Mahapatra

**Affiliations:** ^1^Project Concern International, New Delhi, India; ^2^Bihar Technical Support Unit, Patna, India; ^3^Bihar Rural Livelihoods Promotion Society, Bihar, India; ^4^Independent Researcher, Ranchi, India

**Keywords:** self-help groups, JEEViKA technical support program, maternal health, newborn care, child nutrition, social development, women empowerment

## Abstract

**Introduction:**

Bihar Rural Livelihoods Promotion Society launched the JEEViKA program in 2007 to improve livelihoods through the Self-Help Group (SHG) platform. Women’s SHGs have shown members’ health improvements by promoting awareness, practices and access to services. This study investigates whether Health & Nutrition (HN) interventions delivered by JEEViKA Technical Support Program (JTSP) via SHG platforms could improve maternal and newborn health and nutritional behaviors in rural Bihar.

**Methods:**

Annual Household Survey and Married Women of Reproductive Age (MWRA) studies of Bihar Technical Support Unit were used to analyze the effectiveness of JTSP on HN behaviors for mother and their infants in Bihar during 2016–21. Descriptive analysis followed by multivariable (binary and multinomial) logistic regressions were conducted to determine the distribution of and associations between various individual/community and programmatic exposures and outcomes of interest.

**Results:**

During 2016–2021, in Bihar, statewide increase (32 to 47%) in SHG membership across all population strata and expansion of HN layering of JTSP from 101 to 349 blocks corroborated with improvements in Maternal-Newborn-Child Health & Nutrition (MNCHN) indicators in JTSP blocks and SHG members. Substantial increase was observed in ≥3ANC visit (9% points), institutional delivery (10%), skin-to-skin-care (17%), dry cord-care (23%), early initiation of breastfeeding (19%) & complementary feeding (9%). Adjusting for socio-demographic factors and Front-Line Workers’ (FLWs’) advice/counseling, multivariable logistic regression revealed that SHG member in JTSP blocks delivering post-intervention (2021) were more likely (vs 2016) to practice: ≥3ANC visits (Adjusted Odds Ratio: aOR = 1.48, *p* < 0.0001), institutional delivery (aOR = 1.71, *p* < 0.0001), skin-to-skin care (aOR = 3.16, *p* < 0.0001) and dry cord-care (aOR = 2.64, *p* < 0.0001), early initiation of breastfeeding (aOR = 1.61, *p* < 0.0001), complementary feeding (aOR_6-8 months_ = 1.48, *p* < 0.0001) and minimum dietary diversity (aOR_6-8 months_ = 1.24). Better mobility, decision making, economic independence and overall empowerment were also evident among SHG member MWRA as opposed to non-members after both phases.

**Discussion:**

The results highlight successful HN integration in JEEViKA by JTSP, demonstrating its effectiveness in integrating with State Rural Livelihoods Mission community platforms. JTSP showcases collaboration within a government system and emphasizes systematic introduction and strengthening at multiple levels. This integration has enabled JEEViKA systems to self-sustain its own HN implementation processes, paving the way for cross-sectoral comprehensive delivery mechanisms for social development.

## Introduction

There has been substantial evidence suggesting economic growth with the incorporation of a well-developed and inclusive financial system, which in turn reduces income inequality and poverty (1). With the concept of financial system for poor, India’s Self-Help Group (SHG) movement started over 30 years ago using micro-financing as a tool to alleviate poverty and to empower women via financial inclusion. Since then it has emerged as the world’s largest women-owned community-based microfinance institution (2, 3). Similar groups based on the development of micro-finance institutions have been found in Kenya (4), Nigeria (5), Ghana (6, 7), Guatemala (7), and South East Asia (8). The concept of SHGs in India involves informal groups of 10–20 women having similar socio-economic background and living in close proximity. They come together for mutual aid and benefit with sources of finance via non-government organizations (NGOs) and nationalized banks (2, 9). Each member of the SHG contributes an amount of Rs 10 to Rs 100 to be deposited in the bank, and basis that the group can obtain loans from the bank out of their own funds (8). The SHG comprises of 2–3 elected leaders in the group, who maintain simple accounts of this collected money and given loans (10). The members conduct regular meetings of the SHG at periodic intervals, mostly once in a month (11). These microfinance institutions that offer small loans for self-employment help enhance livelihoods and quality of life (12–14). Several studies have shown the positive impact of SHGs on women’s economic, social and political aspects (15–19). In developing nations, SHG membership is being promoted to improve access to credit and mobilize microfinancing (20). These women SHG-members come from marginalized and economically disadvantaged background, mostly rural, with minimal or no land ownership, low literacy levels, and lack agency on their own health and financial aspects (3, 21). The majority of the SHG women belong to the middle age group (36–50 years) (22–25). Several studies from India have investigated the factors influencing SHG membership, and have found that SHG membership tends to increase with the age of women, which might be attributed toward the greater social mobility for women in traditional settings as they take on caregiving roles within their families (26–29).

Research demonstrates women’s SHGs as an opportunity to health improvement by increasing knowledge on healthy practices in the community that leads to behavioral changes, and by enhancing access to health-related services to the poor and marginalized communities through addressing financial, geographic and other barriers (30–33). In 1970, a community development initiative in Jamkhed, Maharashtra, India, used participatory approach, identified and trained women as health workers and provided with funds for health emergencies. Over the first 20 years, this program significantly improved health outcomes with infant mortality rates dropping from 176 to 19 per 1,000 live births, and the birth rate falling from 40 to 20 per 1,000 people. Access to antenatal care, safe delivery, and immunization became nearly universal, while malnutrition rates decreased from 40% to under 5% (34, 35). Another study on integrating a micro-credit forum with family planning and immunization programs revealed that membership in the forum positively impacted maternal knowledge of prenatal care, led to increased use of contraceptives, and contributed to a decline in fertility rates (36). Likewise, in a village in rural India, presence of SHG have shown increased knowledge of family planning and maternal health service uptake in the community (37). A systematic review of randomized controlled trials demonstrated the positive impact of community based women’s groups on neonatal mortality across socioeconomic strata in a multi-country meta-analysis (38). Evidence also suggests significant impact of group-based nutrition behavior change communication (BCC) interventions on maternal and child nutrition and feeding practices as well as hygiene behavior, addressing undernutrition and health practices in the community (39–41). While substantial evidence indicates that interventions involving women’s groups have a positive impact on health, questions remain about the coverage and effectiveness of these approaches when implemented on a larger scale. In addition, mixed results in the outcome have been common as well while implementing health interventions via SHGs, involving no significant impact on some outcome variables (42, 43). But with the reach and scale of SHG platforms, these studies indeed suggest the potential of these women groups to effectively extend the impact of thematic interventions by reaching more women and their families to encourage positive behaviors, leading to better outcomes.

With the vision of social & economic empowerment of the rural poor in the state of Bihar (India), Bihar Rural Livelihoods Promotion Society (BRLPS) under jurisdiction of the state Government of Bihar, with support from the World Bank, launched an ambitious program known as JEEViKA (meaning *“livelihood”*). The aim was to mobilize women through SHGs in rural Bihar and empower them with strategies to improve livelihoods and economic security. It started in the year of 2007 in 6 blocks of 6 districts of Bihar with plan for scale up to the entire state, and by 2014, JEEViKA had already formed around 350,000 SHGs across all the districts of Bihar (44, 45). The key purpose of Jeevika was to bring socio-economic change in rural Bihar, by mobilizing women from impoverished households into SHGs and then delivering targeted funds for credit, food security, health emergencies, and livelihood opportunities (46, 47). In the early stages of the Jeevika intervention, evidence from a randomized controlled trial had observed a reduction in the debt and asset build up in the SHG (46).

Though the primary purpose of SHGs has been to economically empower women and communities, but with its broad population coverage, the platform has also been sought after to deliver development by layering it with various thematic interventions (48). Thus, considering health interventions layering onto SHG platforms, an NGO (Project Concern International (PCI)) led pilot project called Parivartan (meaning *“transformation”*) was implemented in 2011 funded by the Bill & Melinda Gates Foundation (BMGF). The strategic objectives were to influence specific maternal, newborn, & child Health, Nutrition and Sanitation (HNS) behaviors among women of reproductive age from the most marginalized communities in 8 programmatically prioritized districts in Bihar, by forming its own SHGs, and using this platform to promote healthy behaviors via community mobilization (45). The conceptual success of the Parivartan project and the need to address health and nutritional issues of women and children in an improved livelihood scenario with better economic stability, led to the idea of leveraging the SHG platform via JEEViKA at a larger scale to improve the maternal and child health and nutritional outcomes in the state. Thus in 2014, to test the feasibility of similar (Parivartan-like) interventions among JEEViKA groups and to learn how to work with the JEEViKA groups before suggesting a significant scale-up of HNS integration within the JEEViKA network, the Parivartan project was expanded to 9 more blocks with existing JEEViKA SHG networks. Findings from an evaluation showed encouraging results across most RMNCH indicators in the Parivartan intervention (49). It observed improved use of contraceptive methods, institutional delivery, skin-to-skin care, delayed bathing, timely initiation and exclusive breastfeeding, age-appropriate immunization among the SHG women as opposed to non-SHG (31). Similarly, another randomized controlled trial on a pilot intervention of health and nutrition BCC via Jeevika SHGs has demonstrated significant impacts on women and child dietary diversity, and consumption of iron–folic acid (IFA) tablets and calcium tablets for pregnant women, using strategic pathways of delivering key messages through the SHG platform and enhancing the knowledge on health and nutritional aspects for women (50). Empowerment being a key conceptual idea of JEEViKA’s SHG platforms, several defining components of women empowerment such as mobility, decision making and economic independence were also within the pursuit of the interventions among women of reproductive age (15–49 years).

Ultimately, with the vision of layering HNS onto JEEViKA SHG network, a government-led initiative was conceptualized to provide technical support to JEEViKA by PCI in 2015, called JEEViKA Technical Support Program (JTSP). It started with the goals to provide better quality of life in terms of improved health, nutrition, and sanitation outcomes among the poorest and most marginalized populations (with a special focus on women and children) in Bihar. Objectives were to integrate health and nutrition within JEEViKA program’s mandate; and to drive innovation and evidence-based approaches and capacity within JEEViKA to scale-up through their SHGs across Bihar. The first phase of JTSP took place between 2015 and 2018 in 101 blocks of 11 districts, with the layering of HNS interventions to JEEViKA at the state, district and block levels. During phase I, JTSP piloted a multi-touch point integrated HNS package, with three main interventions. SHG-level monthly roll out of 15 HNS themes were conducted, wherein the first weekly SHG meeting was designated for HNS activities, in which the community mobilizer would interact with the SHG members to build an environment, consensus and awareness on the HNS theme. Household level visits of target beneficiaries by designated members of the village organization (VO) called the health subcommittees (HSC), where JTSP developed tools to reinforce key messages and help the target beneficiary adopt relevant health and nutrition practices. Community based awareness events focused on various themes such as exclusive breastfeeding, diarrhea prevention and management, as well as complementary feeding.

**Figure 1:**
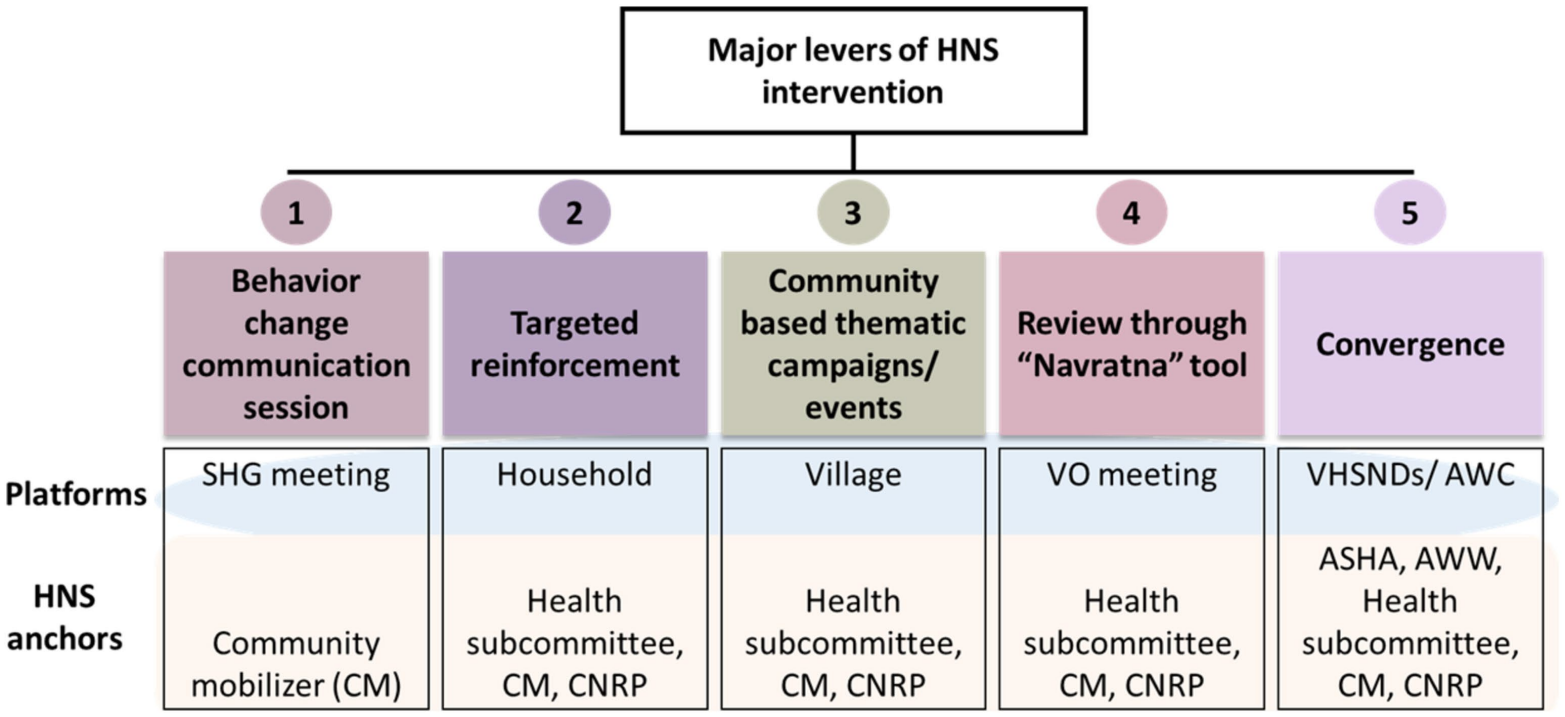
Five major levers of HNS intervention of JEEViKA technical support program (CNRP, Community Nutrition Resource Person).

**Figure 2:**
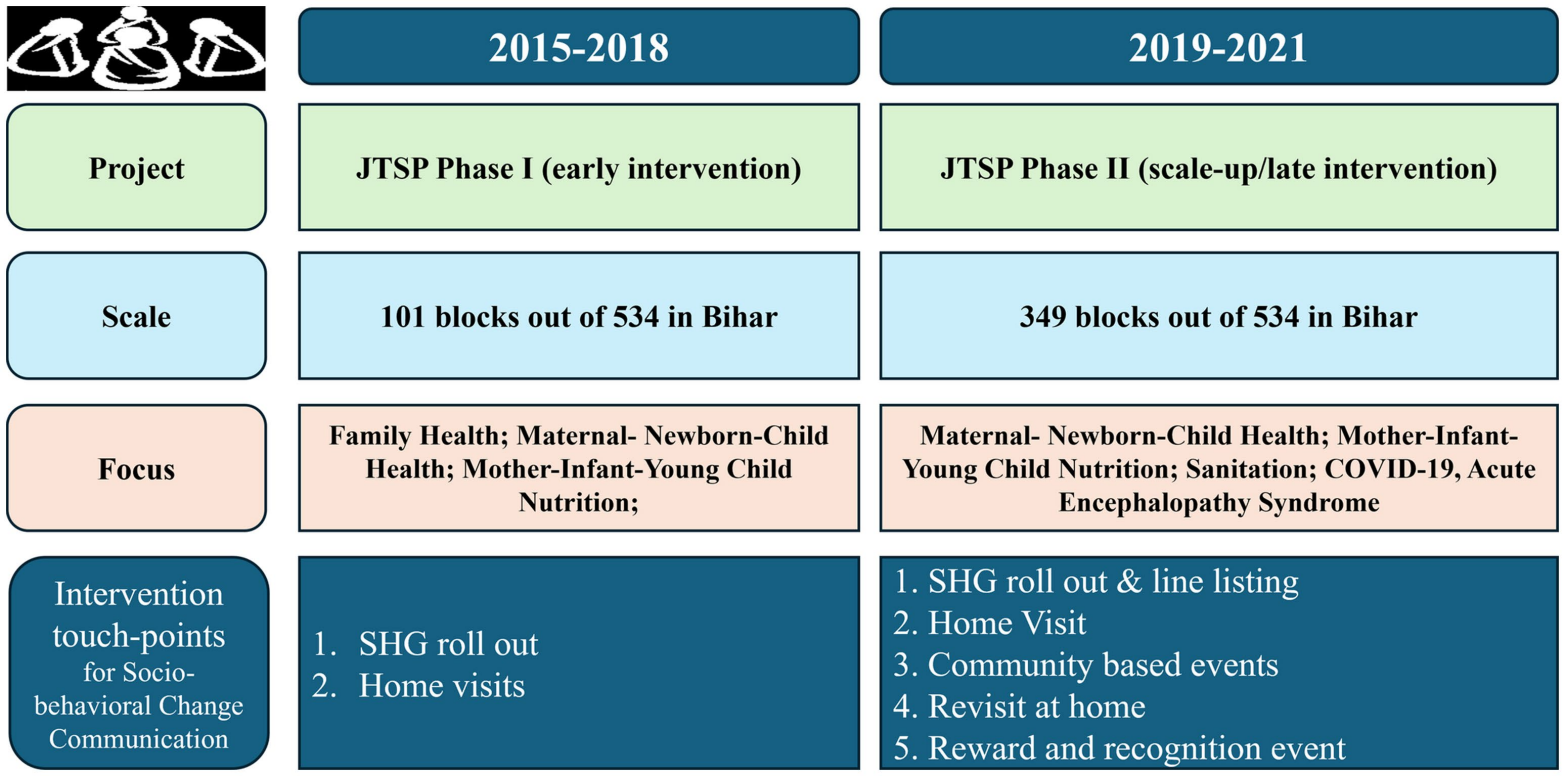
The evolution of JTSP through phases.

Further, the second phase of JTSP initiated with a scale-up in 2018 and ended in 2020, during which the HNS interventions were expanded to 349 blocks of 35 districts of Bihar. During this phase, HNS became a mandate to JEEViKA, making all the related entities and people responsible for HNS along with JEEViKA’s economic empowerment and livelihood programs. By 2018, a dedicated community cadre for Health and Nutrition (HN) had been introduced in the system, comprising of the Master Resource Person (MRP) at cluster level, and the Community Nutrition Resource Person (CNRP) at the panchayat level. The strategy of inviting Front-Line Workers (FLWs) to attend meetings and events helped them better engage in community-level campaigns. The evolution of JTSP’s supportive work with JEEViKA led to the development of a multi-pronged behavior change intervention package (with multiple exposures) which was rolled out across Bihar, through JEEViKA community-based organizations as shown in Figure 1. The evolution of JTSP across phases through its expansion and modifications are explained in Figure 2.

This paper presents the findings from studies conducted by Bihar Technical Support Unit (BTSU), as part of a decade long statewide system strengthening effort, independent of the JTSP implementation. We hypothesized that HN interventions delivered with the support of JTSP via JEEViKA’s SHG platforms would help in improving healthy behaviors for mother and newborn in rural communities of Bihar, thus emphasizing the importance of a technical support program to deliver additional strategies/interventions from different sectors using ready to implement government-based platforms. To test this hypothesis there was a need to analyze program monitoring data generated independent of JTSP intervention, to investigate whether health & nutrition (HN) interventions delivered by JEEViKA Technical Support Program (JTSP) via SHG platforms could improve maternal and newborn health and nutritional behaviors in rural Bihar.

## Materials and methods

### Measures: key outcomes and covariates

The SHG membership was considered among those households where either recently delivered women (mothers of children aged 0–11 months) or any other women of that household was a member of SHG.

The key outcomes were considered under three domains - maternal health (ANC clinic visits and institutional delivery), newborn care (skin-to-skin care, dry cord-care, early initiation of breastfeeding) for children aged 0 to 2 months, and child nutrition (initiation of complementary feeding, minimum acceptable diet, and minimum dietary diversity) for children aged 6 to 8 and 9 to 11 months.

Mothers of infants aged 0–2 months were enquired about the number of ANC clinic visits (to determine any, ≥3 & ≥4 ANC visit) made during their last pregnancy. Institutional delivery was defined as whether the mother of 0 to 2 months-old infant delivered her youngest child at a health facility (institution) or not. Whether or not the mother of 0–2 months-old infant had her baby kept naked on her chest, next to her skin immediately after delivering the baby constituted the practice of skin-to-skin care. The practice of dry cord-care was measured based on whether ‘anything’ or ‘nothing’ was applied to the cord immediately after cutting or later, until the cord fell off. The initiation of breast-feeding within an hour after birth was defined as early. All indicators of nutrition were defined based on IYCF guidelines and standard definitions. Timely initiation of complementary feeding was measured based on whether anything other than milk, water or medicine was fed to the infant after completing 6 months of age. For infants aged 6 to 8 months and 9 to 11 months, dietary diversity and minimum acceptable diet were measured using 24-h recall data. Whether or not the infant was given greater than or equal to four out of seven food groups constituted minimum dietary diversity. Minimum acceptable diet comprised of minimum dietary diversity as well as whether solid/semi-solid/soft food was given to the infant aged 6–8 months at least twice a day and to infants aged 9–11 months at least thrice a day.

To measure the impact of the program beyond health, indicators of women empowerment - mobility, economic independence, and women’s participation in household decisions were included in the analysis. Responses to individual items under each of the three components were summed up to get an aggregate score which was categorized into “poor,” “average,” “good” based on tertile boundaries to measure overall level of women empowerment, along with individual component indicators of women empowerment.

Known socio-demographic indicators of health inequities and factors known to affect healthcare utilization were measured as potential confounders. This included mother’s age, parity, religion, caste, education (years of formal education of the mother collected as a continuous variable and was adjusted in the regression models) and wealth tertile (based on tertile distribution of multi-components, pre-validated log-transformed asset index).

Led by a not for profit, non-governmental organization: CARE India Solutions for Sustainable Development, BTSU was functional since 2013, in all 38 districts of Bihar and was working closely with the State Government of Bihar under financial patronage of the Bill and Melinda Gates Foundation. As a Technical Support Unit, BTSU provided catalytic support to the Health and Social Welfare Departments of Government of Bihar (GoB) for systems strengthening in maternal and child health, family planning and nutrition interventions. The Concurrent Measurement and Learning (CML) Unit of BTSU conducted regular data collection on an ongoing basis to inform programs. In the present piece of work, data from Annual Household survey and Married Women of Reproductive Age (MWRA) study have been used to analyze the effectiveness of JTSP in Bihar for HN layering in SHGs during 2016–21. Using multistage cluster random sampling with systematic random sampling at the household selection with a random start, the studies used as the data source for this analysis did recruit a statewide representative sample of rural Bihar (for MWRA study it was both urban and rural Bihar).

### Study design, participants and sampling strategy

#### The household survey

Several rounds of household surveys, concurrent with program implementation timelines, were conducted across Bihar, by BTSU, during 2011–2021 to assess changes in key HN indicators or healthy practices promoted via various interventions in the state. In these surveys, recently delivered mothers of children aged 0 to 2, 3 to 5, 6 to 8, 9 to 11, and 12 to 23 months were interviewed to understand their health-related practices for themselves during pregnancy, neonatal care, child nutrition, and immunization. In the present work, data from 2016 to 2021 have been analyzed.

Sample size was estimated using binomial formula for a proportion with finite population correction: *n* = [p(1-p) *N]/ [N*D + p (1-p)]; where *p* = expected proportion (here 0.5 was used to be most conservative); N = Total population size; D = B^2^/(Zα/2)^2^; where: B is half of the width of the desired confidence interval.

The surveys included samples from all blocks of all districts (38) of Bihar. The required number of Anganwadi Centers (AWCs: based on sample size calculation and least possible number per block being 19) were randomly sampled from the list of all AWCs per block and 1 sample (consisting of one child from each of the five age groups) per selected AWC selected systematically. In each selected AWC, after selecting the index house randomly, following a right hand rule (always move to right), households are selected with an interval of five to recruit the next eligible mother/child. Only one child was recruited in each household and the mother was interviewed. Thus, a total of 78,435 mothers (15,687 from each age group) were finally interviewed.

#### Survey of married women of reproductive age

It was a large survey across all 38 districts of Bihar conducted in multiple iterations in 2016, 2018, and 2021 to capture a comprehensive understanding of family planning interventions, behavior and practices from Married Women of reproductive age 15 to 49 years. The survey employed a multi-stage, stratified probability sampling technique in three stages - district, block and AWC/Ward. The sample size was calculated following the binomial formula: Assuming an *α* error of 5%, *β* error of 20% (power = 0.8) and absolute precision of 10%, the desired sample size for each district turned out to be 384 which got inflated to 576 after incorporating a design effect of 1.5. To account for 2–4% data loss, a rounded figure of 600 per district was decided upon.

To recruit the participants for the interview, 5 blocks and 120 AWCs were selected based on proportional allocation from each of the 38 districts. ‘Buildings’ or ‘structures’ containing human dwellings were identified from the sampled AWCs (for rural areas) and Wards (for urban areas) for conducting the interviews. Thus, the total sample of 22,800 (600*38 districts; MWRA) respondents were recruited and interviewed from the entire state during 2016 and 2018 while in 2021, 22,668 MWRAs were interviewed.

Reach of JTSP in the community via women’s SHG platform and the effectiveness of HN interventions through these platforms on maternal, newborn and child health behaviors were determined using a representative sample of mothers with children aged 0–11 months in JTSP blocks of rural Bihar during Phase I and II.

Given that the JTSP program was ongoing in 101 blocks during 2016 and scaled-up to 349 blocks during 2018, the responses from participants of these blocks were analyzed to assess the program effectiveness on outcome indicators of interest (programmatically relevant to JTSP) using the samples of mothers having babies belonging to following 3 age groups: 0 to 2, 6 to 8, and 9 to 11 months from HHS.

The interviews were conducted using pre-tested structured digital questionnaires in Hindi language.

### Statistical analysis

Descriptive [frequency, proportions and the corresponding 95% confidence Intervals (95% CIs)] analysis was conducted to determine the distribution of various parameters in the study population. Multivariable logistic regressions were conducted further to determine the associations between various individual, community and program related predictors and outcomes of interest, adjusting for potential confounders (respondents’ age, religion, caste, education and wealth-tertile as well as corresponding FLW advice/counseling on specific practices). All analyses were conducted using SAS version 9.4.

### Ethics approval

The study protocols and procedures were reviewed and approved by the Ashirwad Ethics Committee, Ashirwad Hospital & Research Center, Ulhasnagar, India. Verbal informed consent was obtained from each agreeing participant before the interview, after explaining the details of the study in the local language.

## Results

Table 1 presents the expansion of SHG membership state-wide and in JTSP blocks during 2016 to 2021, describing the reach of JTSP among households of recently delivered women. At state level, the SHG membership increased from 32 to 47% in this period. In the 101 JTSP phase I blocks, the proportion of households with SHG members increased from 39% in 2016 to 46% in 2018. During the JTSP scale-up phase during 2018 to 2021, in 349 blocks, the households with SHG membership increased from 42% to ~48%.

**Table 1:** Reach of SHG membership during the duration of JEEViKA Technical Support Program (JTSP) among families of recently delivered (in last 1 year) women in Bihar.

Year	Statewide (All 534 blocks)			Phase I (101 JTSP blocks)			Phase II (349 JTSP blocks)		
	N	N	Percent (95% CI)	N	n	Percent (95% CI)	N	n	Percent (95% CI)
2016	62,667	20,832	32.0 (31.6–32.4)	11,184	4,482	38.6 (37.6–39.6)	–		
2017	62,748	25,198	38.6 (38.2–39.0)	11,184	5,052	44.0 (43.0–45.0)			
2018	62,748	27,819	42.7 (42.3–43.2)	10,044	4,775	46.1 (45.1–47.2)	41,508	18,318	42.0 (41.5–42.6)
2019	62,748	27,830	42.9 (42.4–43.3)	–			41,508	18,392	42.6 (42.0–43.1)
2020	62,748	31,218	48.6 (48.2–49.0)				41,508	20,724	48.3 (47.8–48.9)
2021	62,748	30,392	47.4 (46.9–47.8)				41,507	20,161	47.2 (46.6–47.7)

N refers to the total sample of women who delivered in last one year across different sets of blocks in Bihar in specific years. n refers to the total number of women from SHG families, who delivered in last one year across different sets of blocks in Bihar in specific years.

Tables 2, 3 present the distribution of the participants and SHG households, across sociodemographic strata. Majority (both overall and in SHG households) were aged between 20 to 30 years, Hindu and multiparous. The distribution of SHG membership across sociodemographic strata revealed that during both Phases, I (2016 to 2018) and II (2018–2021), the membership increased substantially among recently delivered women as well as women of reproductive age (15–49 years) of all categories of age, parity, religion, wealth tertile/economic backgrounds and also among non-marginalized in JTSP blocks. During scale-up phase from 2018 to 2021, there has been slight increase (4–6%) in SHG membership of women belonging to all categories of ages, parity, religion (both Hindu & non-Hindu), caste (both marginalized & non-marginalized) and economic backgrounds.

**Table 2:** SHG membership across sociodemographic strata among mothers of children aged 0–11 months in JTSP blocks of Bihar (2016–2021).

Sociodemographic strata		Phase I (101 blocks)						Phase II (Scale up: 349 blocks)					
		2016 (*N* = 11,184)			2018 (*N* = 10,044)			2018 (*N* = 41,508)			2021 (*N* = 41,507)		
		N	SHG membership (4482)		N	SHG membership (4775)		N	SHG membership (18318)		N	SHG membership (20161)	
			n	Percent (95% CI)		n	Percent (95% CI)		n	Percent (95% CI)		N	Percent (95% CI)
Age (in years)	Below 20	1,285	527	11.8 (10.8–12.7)	1,023	498	10.4 (9.6–11.3)	3,593	1,662	9.1 (8.7–9.5)	3,242	1,575	7.8 (7.4–8.2)
	20–30	9,099	3,607	80.5 (79.3–81.6)	8,459	3,991	83.6 (82.5–84.6)	35,279	15,458	84.4 (83.9–84.9)	35,750	17,307	85.8 (85.4–86.3)
	31–40	378	178	4.0 (3.4–4.5)	308	151	3.2 (2.7–3.7)	1,362	629	3.4 (3.2–3.7)	1,325	660	3.3 (3.0–3.5)
	More than 40	422	170	3.8 (3.2–4.4)	254	135	2.8 (2.4–3.3)	1,274	569	3.1 (2.9–3.4)	1,190	619	3.1 (2.8–3.3)
Parity*	0 or 1	3,258	1,233	27.6 (26.3–28.9)	2,924	1,310	27.4 (26.2–28.7)	12,620	5,181	28.3 (27.6–28.9)	12,089	5,374	26.7 (26–27.3)
	2	3,146	1,173	26.2 (24.9–27.5)	2,861	1,277	26.7 (25.5–28.0)	11,812	4,958	27.1 (26.4–27.7)	12,947	6,003	29.8 (29.1–30.4)
	More than 2	4,748	2066	46.2 (44.7–47.7)	4,259	2,188	45.8 (44.4–47.2)	17,076	8,179	44.7 (43.9–45.4)	16,471	8,784	43.6 (42.9–44.3)
Religion	Hindu	9,880	4,085	91.1 (90.3–92)	8,990	4,372	91.6 (90.8–92.3)	36,188	16,635	90.8 (90.4–91.2)	36,104	18,272	90.6 (90.2–91.0)
	Non-Hindu	1,304	397	8.9 (8.0–9.7)	1,054	403	8.4 (7.7–9.2)	5,320	1,683	9.2 (8.8–9.6)	5,403	1889	9.4 (9.0–9.8)
Caste	Marginalized	3,108	1,543	34.4 (33–35.8)	2,871	1,532	32.1 (30.8–33.4)	11,207	5,872	32.1 (31.4–32.7)	10,980	6,021	29.9 (29.2–30.5)
	Non-marginalized	8,076	2,939	65.6 (64.2–67.0)	7,173	3,243	67.9 (66.6–69.2)	30,301	12,446	67.9 (67.3–68.6)	30,527	14,140	70.1 (69.5–70.8)
Wealth tertile	Lower	3,883	1,560	34.8 (33.4–36.2)	3,286	1,469	30.8 (29.5–32.1)	12,617	5,540	30.2 (29.6–30.9)	12,942	6,251	31.0 (30.4–31.6)
	Middle	4,032	1,677	37.4 (36.0–38.8)	3,253	1,659	34.7 (33.4–36.1)	13,990	6,593	36.0 (35.3–36.7)	13,984	7,164	35.5 (34.9–36.2)
	Upper	3,269	1,245	27.8 (26.5–29.1)	3,505	1,647	34.5 (33.1–35.8)	14,901	6,185	33.8 (33.1–34.4)	14,581	6,746	33.5 (32.8–34.1)

*Parity information was available for 11,152 participants in 2016. N refers to the total number of mothers of children aged 0–11 months in JTSP blocks of Bihar in specific years or in specific sociodemographic strata. n refers to the number of mothers of children aged 0–11 months of SHG families in JTSP blocks of Bihar in specific years, in specific sociodemographic strata.

**Table 3:** SHG membership across sociodemographic strata among married women of reproductive age (15 to 49 years) in JTSP blocks of Bihar (2016–2021).

Sociodemographic strata		Phase I						Phase II (Scale up)					
		2016 (*N* = 4,319)			2018 (*N* = 3,645)			2018 (*N* = 15,771)			2021 (*N* = 15,223)		
		N	SHG membership (1001)		N	SHG membership (1412)		N	SHG membership (5124)		N	SHG membership (5731)	
			n	Percent (95% CI)		n	Percent (95% CI)		n	Percent (95% CI)		N	Percent (95% CI)
Age (in years)	Below 20	281	21	2.1 (1.2–3.0)	312	43	3.0 (2.1–3.9)	1,172	142	2.8 (2.3–3.2)	760	78	1.4 (1.1–1.7)
	20–30	2,170	451	45.1 (42.0–48.1)	1852	671	47.5 (44.9–50.1)	7,808	2,332	45.5 (44.1–46.9)	7,128	2,252	39.3 (38.0–40.6)
	31–40	585	162	16.2 (13.9–18.5)	488	240	17.0 (15.0–19.0)	2075	844	16.5 (15.5–17.5)	1972	889	15.5 (14.6–16.4)
	More than 40	1,283	367	36.7 (33.7–39.6)	993	458	32.4 (30.0–34.9)	4,716	1806	35.2 (33.9–36.6)	5,363	2,512	43.8 (42.5–45.1)
Parity	0 or 1	829	78	7.8 (6.1–9.5)	693	95	6.7 (5.4–8.0)	3,103	389	7.6 (6.9–8.3)	2,812	431	7.5 (6.8–8.2)
	2	804	144	14.4 (12.2–16.6)	723	243	17.2 (15.2–19.2)	3,142	838	16.4 (15.3–17.4)	3,390	996	17.4 (16.4–18.4)
	More than 2	2,686	779	77.8 (75.2–80.4)	2,229	1,074	76.1 (73.8–78.3)	9,526	3,897	76.1 (74.9–77.2)	9,021	4,304	75.1 (74–76.2)
Religion	Hindu	3,819	922	92.1 (90.4–93.8)	3,216	1,275	90.3 (88.8–91.8)	13,627	4,631	90.4 (89.6–91.2)	13,425	5,185	90.5 (89.7–91.2)
	Non-Hindu	500	79	7.9 (6.2–9.6)	429	137	9.7 (8.2–11.2)	2,144	493	9.6 (8.8–10.4)	1798	546	9.5 (8.8–10.3)
Caste	Marginalized	1,191	352	35.2 (32.2–38.1)	871	388	27.5 (25.2–29.8)	3,947	1,662	32.4 (31.2–33.7)	2,983	1,446	25.2 (24.1–26.4)
	Non-marginalized	3,128	649	64.8 (61.9–67.8)	2,774	1,024	72.5 (70.2–74.8)	11,824	3,462	67.6 (66.3–68.8)	12,240	4,285	74.8 (73.6–75.9)
Wealth tertile	Lower	1,383	362	36.2 (33.2–39.1)	1,289	552	39.1 (36.5–41.6)	4,881	1894	37.0 (35.6–38.3)	4,757	2,187	38.2 (36.9–39.4)
	Middle	1,525	398	39.8 (36.7–42.8)	1,211	514	36.4 (33.9–38.9)	5,337	1948	38.0 (36.7–39.3)	5,199	2,176	38.0 (36.7–39.2)
	Upper	1,411	241	24.1 (21.4–26.7)	1,145	346	24.5 (22.3–26.7)	5,553	1,282	25.0 (23.8–26.2)	5,267	1,368	23.9 (22.8–25.0)

N refers to the total number of married women of reproductive age (15 to 49 years) in JTSP blocks of Bihar in specific years or in specific sociodemographic strata. n refers to the number of married women of reproductive age (15 to 49 years) of SHG families in JTSP blocks of Bihar in specific years, in specific sociodemographic strata.

During JTSP phases, corroborating rise in several Maternal-Newborn-& Child Health & Nutrition (MNCHN) indicators signified the uptake of recommended practices related to health and nutrition by the recently delivered women with children aged 0–11 months. Pre-post comparison for Phase I (2016 vs. 2018) revealed increased ANC visit by pregnant women in JTSP-Phase I blocks (101): 42 to 53% for ≥3 and 23 to 28% for ≥4 visits. The institutional delivery increased in JTSP blocks from 73 to 80%. During the same phase, in those blocks, the practice of newborn care with indicators such as skin-to-skin care, dry cord-care and early initiation of breastfeeding also improved to 50, 51 and 74% from 36, 44, and 65%, respectively. The nutritional practices by mothers improved showing a 13 and 11% rise in initiation of complementary feeding for children aged 6–8 months and 9–11 months respectively, whereas, the practices almost doubled for minimum dietary diversity (6 to 10% for 6–8 months and 14 to 24% for 9–11 months) and minimum acceptable diet (5 to 9% for 6–8 months and 11 to 18% for 9–11 months) in both the age groups.

Post-Phase II, in JTSP-Phase II blocks (349): improvement was observed in ANC visits for ≥3 (51 to 62%) and ≥ 4 visits (29 to 36%) by the pregnant women from 2016 to 2021. A 10% rise in institutional delivery was observed during the span. Remarkable rises in newborn care practices such as skin-to-skin care (32 to 59%), dry cord-care (41 to 64%) and early initiation of breastfeeding (62 to 71%) were also recorded during the scale-up phase. The nutritional practices improved with initiation of complementary feeding for both children aged 6–8 months (51 to 60%) and 9–11 months (74 to 84%). The minimum dietary diversity (14 to 17%) and minimum acceptable diet (11 to 14%) also increased for children aged 9–11 months (Table 4).

**Table 4:** Uptake of recommended MNCHN practices by mothers of children aged 0–2 months, 6–9 months and 9–11 months across JTSP blocks pre-post phase 1 and phase II (Scale-up).

Practices	Age category (months)	Phase I (101 blocks)						Phase II (Scale up: 349 blocks)					
		2016			2018			2016			2021		
		N	Doing recommended practice		N	Doing recommended practice		N	Doing recommended practice		N	Doing recommended practice	
			n	Percent (95%CI)		n	Percent (95%CI)		n	Percent (95%CI)		n	Percent (95%CI)
Any visit to ANC clinic	0 to 2	2,796	2,689	96.2 (95.4–96.9)	2,505	2,430	97.3 (96.6–97.9)	10,376	10,122	97.5 (97.2–97.8)	10,364	10,175	98.2 (97.9–98.5)
≥3 visit to ANC clinic		2,773	1,125	41.8 (39.9–43.8)	2,430	1,258	52.5 (50.3–54.6)	10,309	5,183	50.8 (49.7–51.9)	10,175	6,286	62.2 (61.1–63.2)
≥4 visit to ANC clinic		2,773	607	23.3 (21.6–25.1)	2,430	657	27.6 (25.6–29.5)	10,309	2,870	28.7 (27.7–29.7)	10,175	3,631	36.4 (35.3–37.5)
Institutional delivery		2,796	2038	73.0 (71.2–74.8)	2,511	2018	80.3 (78.6–82.0)	10,376	7,589	72.5 (71.5–73.5)	10,377	8,603	82.9 (82.0–83.7)
Skin-to-skin care		2,561	941	35.9 (33.9–37.9)	2,257	1,122	49.6 (47.4–51.8)	9,322	2,923	32.1 (31.0–33.1)	9,170	5,507	59.3 (58.2–60.5)
Dry cord-care		2,346	1,067	44.0 (41.8–46.1)	2,183	1,109	50.9 (48.6–53.1)	8,856	3,683	40.9 (39.7–42.0)	9,157	5,909	64.1 (62.9–65.2)
Early initiation of breastfeeding		2,796	1798	64.8 (62.9–66.6)	2,511	1842	73.7 (71.9–75.5)	10,376	6,383	61.8 (60.7–62.8)	10,377	7,329	70.7 (69.8–71.7)
Exclusively breastfed in last 24 h		2,780	2,264	82.4 (80.9–83.9)	2,511	2035	81.0 (79.4–82.7)	10,275	8,051	79.6 (78.8–80.5)	10,377	8,327	81.0 (80.2–81.8)
Initiation of complementary feeding	6 to 8	2,796	1,257	46.6 (44.6–48.6)	2,511	1,437	59.5 (57.4–61.5)	10,376	5,266	50.6 (49.6–51.7)	10,377	6,171	60.0 (59.0–61.1)
	9 to 11	2,796	1865	68.8 (67.0–70.6)	2,511	1957	79.8 (78.1–81.4)	10,377	7,676	74.3 (73.4–75.2)	10,377	8,723	84.2 (83.5–85.0)
Minimum dietary diversity in children	6 to 8	2,796	148	5.5 (4.6–6.4)	2,511	248	9.9 (8.6–11.1)	10,376	625	5.7 (5.2–6.2)	10,377	814	7.4 (6.8–7.9)
	9 to 11	2,796	376	13.8 (12.4–15.2)	2,511	582	23.8 (22.0–25.6)	10,377	1,499	14.3 (13.5–15.0)	10,377	1923	17.4 (16.6–18.2)
Minimum acceptable diet	6 to 8	2,796	136	4.9 (4.1–5.8)	2,511	224	9.1 (7.9–10.4)	10,376	543	5.0 (4.5–5.4)	10,377	772	7.0 (6.4–7.5)
	9 to 11	2,796	302	10.7 (9.5–11.9)	2,511	419	17.6 (15.9–19.2)	10,377	1,185	11.1 (10.4–11.8)	10,377	1,596	14.3 (13.5–15.0)

N refers to the total number of mothers of 0–2/6–8/9–11 months old children in JTSP blocks of Bihar in specific years. n refers to the number of mothers of 0–2/6–8/9–11 months old children, who are doing recommended practices in JTSP blocks of Bihar in specific years.

Adjusting for socio-demographic factors such as caste, religion, education, wealth index and parity, as well as FLW advice/counseling on relevant MNCHN practices, it was observed that post JTSP Phase I (in 2018), ANC visits had higher odds of happening for any visit, 3 or more visits, and 4 or more visits, as compared to 2016. Higher odds (aOR = 1.44, *p* < 0.0001) for institutional delivery was also noticed. The newborn care practices were significantly improved for skin-to-skin care, dry cord-care, and early initiation of breastfeeding post JTSP Phase I (*p* < 0.0001). As for nutritional practices, statistically significant positive associations were found for initiation of complementary feeding, minimum dietary diversity and minimum acceptable diet with JTSP Phase I.

Post scale-up in 2021, pregnant women had significantly higher odds of ANC visits as opposed to 2016. The odds of institutional delivery were also higher (aOR = 1.71, *p* < 0.0001). Newborn care practices such as skin-to-skin care (aOR = 3.16, *p* < 0.0001) and dry cord-care (aOR = 2.64, *p* < 0.0001) were practiced more. The early initiation of breastfeeding was also more in 2021 as opposed to 2016. Nutritional practices including initiation of complementary feeding, minimum dietary diversity and minimum acceptable diet for both 6–8 and 9–11 months old children were significantly higher in 2021, compared to 2016 (Table 5).

**Table 5:** Association* of recommended RMNCH practices by recently delivered mothers in JTSP blocks with JTSP intervention [both phase post-JTSP (2018 and 2021) as opposed to pre-intervention (2016)].

	Age category (months)	Phase I	Phase II (Scale up)
MNCHN indicators		Post-intervention (2018) [Ref: Pre (2016)]	Post-intervention (2021) [Ref: Pre (2016)]
		AOR*	AOR*
Made any ANC clinic visit (ref = not visited)	0 to 2	1.36 (1.30–1.42)	1.19 (1.15–1.23)
Made 3 or more ANC clinic visit (ref = not visited)		1.53 (1.51–1.56)	1.48 (1.46–1.49)
Made 4 or more ANC clinic visit (ref = not visited)		1.20 (1.17–1.22)	1.30 (1.29–1.32)
Institutional delivery (ref = no)		1.44 (1.41–1.46)	1.71 (1.69–1.73)
Provided skin-to-skin care (ref = no)		1.80 (1.77–1.83)	3.16 (3.13–3.19)
Provided dry cord-care (ref = no)		1.33 (1.31–1.35)	2.64 (2.62–2.67)
Initiated early on breastfeeding (ref = no)		1.58 (1.55–1.60)	1.61 (1.59–1.62)
Exclusively breastfed in last 24 h (ref = no)		0.92 (0.90–0.94)	1.14 (1.13–1.16)
Initiated on complementary feeding (ref = no)	6 to 8	1.68 (1.66–1.71)	1.48 (1.46–1.49)
	9 to 11	1.83 (1.80–1.86)	1.82 (1.80–1.84)
Received minimum dietary diversity (ref = no)	6 to 8	1.83 (1.78–1.89)	1.24 (1.21–1.26)
	9 to 11	1.87 (1.83–1.91)	1.19 (1.18–1.21)
Received minimum acceptable diet (ref = no)	6 to 8	1.88 (1.82–1.94)	1.37 (1.34–1.39)
	9 to 11	1.72 (1.68–1.76)	1.24 (1.23–1.26)

*Presented as adjusted odds ratios (considered statistically significant as *p* < 0.0001) from binary multivariable logistic regressions adjusted for caste, religion, education, wealth index, parity and FLW advice/counseling.

Certain parameters to measure women empowerment among MWRA (15–49 years) were also compared between members and non-members of SHG in JTSP blocks. At the end of Phase I (2018) in the JTSP blocks, good mobility (52% vs. 37%), decision making (72% vs. 64%), economic independence (13% vs. 6%), and overall women empowerment (51% vs. 32%) were reported by more SHG member MWRA as opposed to their non-member counterparts. Likewise, at the end of Phase II in 2021, good mobility (55% vs. 39%), decision making (62% vs. 56%), economic independence (11% vs. 5%) and overall women empowerment (40% vs. 33%) were reported by more SHG member MWRA as compared to non-member MWRA (Table 6).

**Table 6:** Women empowerment among SHG member (vs. non-members) married women of reproductive age (15-49 years) in JTSP blocks during phase I and phase II (Scale-up).

Characteristic	Category	Phase I – 2018 (*N* = 3,795)				Phase II (Scale-up) – 2021 (*N* = 15,223)			
		SHG members (*N* = 1,461)		Non-SHG members (*N* = 2,334)		SHG members (*N* = 5,731)		Non-SHG members (*N* = 9,492)	
		n	Percent (95% CI)	n	Percent (95% CI)	n	Percent (95% CI)	n	Percent (95% CI)
Mobility	Poor	331	22.7 (20.5–24.8)	994	42.6 (40.6–44.6)	1,478	25.8 (24.7–26.9)	4,214	44.4 (43.4–45.4)
	Average	375	25.7 (23.4–27.9)	479	20.5 (18.9–22.2)	1,126	19.6 (18.6–20.7)	1,550	16.3 (15.6–17.1)
	Good	755	51.7 (49.1–54.2)	861	36.9 (34.9–38.8)	3,127	54.6 (53.3–55.9)	3,728	39.3 (38.3–40.3)
Decision making	Poor	248	17.0 (15.0–18.9)	545	23.4 (21.6–25.1)	1,298	22.6 (21.6–23.7)	2,637	27.8 (26.9–28.7)
	Average	158	10.8 (9.2–12.4)	304	13.0 (11.7–14.4)	911	15.9 (14.9–16.8)	1,540	16.2 (15.5–17.0)
	Good	1,055	72.2 (69.9–74.5)	1,485	63.6 (61.7–65.6)	3,522	61.5 (60.2–62.7)	5,315	56.0 (55.0–57.0)
Economic independence	Poor	59	4.0 (3.0–5.0)	209	9.0 (7.8–10.1)	155	2.7 (2.3–3.1)	662	7.0 (6.5–7.5)
	Average	1,213	83.0 (81.1–85.0)	1988	85.2 (83.7–86.6)	4,959	86.5 (85.6–87.4)	8,383	88.3 (87.7–89.0)
	Good	189	12.9 (11.2–14.7)	137	5.9 (4.9–6.8)	617	10.8 (10.0–11.6)	447	4.7 (4.3–5.1)
Overall empowerment	Poor	138	9.4 (7.9–10.9)	466	20.0 (18.3–21.6)	681	11.9 (11.0–12.7)	2,181	23.0 (22.1–23.8)
	Average	584	40.0 (37.5–42.5)	1,133	48.5 (46.5–50.6)	2,277	39.7 (38.5–41.0)	4,208	44.3 (43.3–45.3)
	Good	739	50.6 (48.0–53.1)	735	31.5 (29.6–33.4)	2,773	39.7 (38.5–41.0)	3,103	32.7 (31.7–33.6)

Multinomial multivariable logistic regression revealed that Post JTSP Phase I in 2018, the SHG member MWRA in JTSP blocks had statistically significant better mobility, decision making, and economic independence as compared to their non-member counterparts. Overall, better empowerment was evidenced among SHG member MWRA as opposed to non-SHG member MWRA. Similarly, post Phase II in 2021, the mobility, decision making and economic independence had significant positive associations with SHG membership among MWRA. Overall, the women empowerment was positively (aOR_average_ = 1.75, *p* < 0.0001; aOR_good_ = 2.73, *p* < 0.0001) associated with SHG membership in JTSP blocks (Table 7).

**Table 7:** Association* of SHG membership with women empowerment among married women of reproductive age (15–49 years) in JTSP blocks during phase I and phase II (Scale-up).

		Phase I (101 blocks) - 2018	Phase II (Scale-up: 349 blocks) – 2021
Indicators	Category	SHG members (Ref: Non-SHG member)	SHG members (Ref: Non-SHG member)
		AOR*	AOR*
Mobility (Ref: Poor)	Average	2.11 (2.04–2.17)	1.87 (1.84–1.90)
	Good	2.28 (2.22–2.34)	2.15 (2.12–2.18)
Decision making (Ref: poor)	Average	1.17 (1.12–1.22)	1.24 (1.22–1.26)
	Good	1.60 (1.55–1.64)	1.44 (1.42–1.46)
Economic independence (Ref: poor)	Average	2.34 (2.22–2.46)	2.69 (2.61–2.77)
	Good	4.77 (4.49–5.08)	5.37 (5.18–5.56)
Empowerment (Ref: poor)	Average	1.74 (1.68–1.80)	1.75 (1.72–1.78)
	Good	3.08 (2.97–3.20)	2.73 (2.68–2.78)

*Presented as adjusted odds ratios (considered statistically significant as *p* < 0.0001) from multinomial multivariable logistic regressions, adjusted for caste, religion, education, wealth index & parity.

Similar positive associations were observed with ordinal logistic regression also (results not shown).

## Discussion

Leveraging upon SHG platforms emerged as one of the major approaches of Indian government underlying the National Rural Livelihood Mission to support women in engaging in livelihood activities and rural development. By 2022, SHGs have already reached out to 79 million households in India and ~ 12 million households in Bihar (51). Though the SHGs primarily are directed toward rural development interventions, but with such an extended reach to households, they also provide a huge platform to reach out to community to deliver additional services, awareness and information (52). The current study also reveals about 47% households (having a mother of an infant) in Bihar (349 blocks) with at least one SHG member in 2021. Thus, these platforms provide a social medium for creating awareness on RMNCH through various strategies with an unprompted community mobilization among SHG members. Furthermore, cross-sectoral approaches are required more to meet the global needs in health and have potential for simultaneous achievement of both economic and health gains (30), and leveraging extensive SHG platforms has been a promising strategy. Several studies have demonstrated the impact of participatory communication with women’s groups on healthy behaviors, safe practices, and better MNCH and nutrition outcomes (31, 49, 53). Such an extending and growing platform provides a number of opportunities to leverage groups as a platform for intervention delivery and reach out to many women at once with resources and information, thus providing a massive coverage. Additionally, integration of HN interventions onto existing SHG platforms also provides an organized structure ready to disperse additional interventions (32). Moreover, such platforms are optimal for behavior change interventions as they provide multiple touch points, involving community, peers and family members thus allowing for ample exposure and dosage of interventions to the target population to bring about a noticeable and sustainable change.

In concurrence to the extensive reach of the SHG platform and its usage for additional health and BCC interventions, our study results also suggested the reach of the program increasing among all strata of population, more among marginalized women, leaving no one behind.

Evidence has suggested that the delivery of development interventions through SHGs could potentially be cost-effective in provision of services at scale. Several studies have shown the maternal and newborn health interventions to be highly cost-effective when delivered through mobilized women’s groups, suggesting that the usage of SHGs for health interventions can lead to cost advantages at a larger scale (54–58). As JEEViKA’s vision was to form approximately 1 million SHGs by 2020, across all blocks in the state of Bihar, JTSP exerted a multi-model approach by providing technical support to JEEViKA to transform lives and improve health outcomes through the social and economic empowerment of poor women and their families participating in the JEEViKA SHGs in Bihar. Such multi-model approach provides an efficient way of introducing several services through one platform with the complementarity of interventions. Sharma et al. (59) has also demonstrated the positive impact of a community-based intervention on both the health and economic outcomes of marginalized women (59). Considering the larger program implementation picture, JTSP enabled JEEViKA to design the Social Behavior Change package, keeping multiple levels of stakeholder influence in mind, not only targeting the individuals for HNS behavior change but also influencing family, community, leaders of community-based organizations and FLWs. The program identified barriers to key HNS behaviors and worked toward them to trigger awareness, action, review and change in practices. Alike others (60), our findings revealed the positive outcome of JTSP interventions with associated increase in uptake of maternal and newborn care practices such as ANC visits, institutional delivery, skin-to-skin care, dry cord-care, and early initiation of breastfeeding in the community. Nutrition related interventions were designed to achieve outcomes related to breastfeeding (early initiation and exclusive) and complementary feeding (dietary diversity in particular) among SHG members. Promising results are shown in the findings with improvement in initiation of complementary feeding, minimum dietary diversity and minimum acceptable diet among children aged 6–11 months during the intervention period (both phases). In line with our study findings, several studies have highlighted the implementation of health interventions via women’s groups with positive outcomes on maternal and newborn health behaviors (37, 42, 49, 57, 61, 62). The systematic plan of determining MNCHN related behavioral practices in the community, analyzing barriers, designing specific interventions, developing key messages toward doable actions, ensuring optimum reach and exposure during implementation, as well as quality probably led to positive outcomes across all domains, thus highlighting the influence of interventions like JTSP in the community.

Another aspect of women’s ability to sustain healthy behaviors for themselves and their children is coupled with their empowerment. Several strategies that can be adopted to empower women include provision of livelihood options, financial independence, encouraging them to compete for leadership positions in the community, gender-equitable division of labor in household, building perceptions of autonomy and self-wellbeing, improving women’s negotiation skills with their normative boundaries, and others (16). Self-confidence, familial and community support help a women to make better and informed decisions toward her own and children’s health (63). SHGs emerged as a platform for women development enabling them to collectively identify the problems in their social and economic environment. Among limited published evidence, a systematic review found that these platforms indeed have positive impact on women’s mobility and economic empowerment (64). Another qualitative study found that access to funds via community platforms did improve women’s independence and decision making (65). This corroborates to the current findings of enhanced empowerment among women SHG members in terms of their mobility, decision making and economic independence and overall empowerment as opposed to non-SHG members.

Alike any other observational study, the current study also had some important limitations. First of all, in the current study the effectiveness of the JTSP program was determined at the population level by comparing practices after the intervention periods (both phases) as opposed to before, not as an experimental study. Owing to the nature of the study being such an observational one, determined associations should not be interpreted as causal, although we tried to minimize the contribution of other pathways of change by controlling for the FLW counseling. This again may have resulted in underestimation of the effectiveness of the program by removing the indirect impact path of JTSP program through FLW channel (as JTSP program induced changes also likely to happen through FLW counseling owing to their engagement with SHGs). Self-reported nature of the practices may also be confounded by indication – those who were practicing may actually be recalling their exposure to FLW counseling better and thus likely to further reduce the magnitude of the associations observed. Despite these potentials for underestimation the substantial magnitude of observed positive associations suggests considerable impact of the JTSP program on the positive deviances in MNCHN practices. The population subsections with better awareness regarding health and nutritional recommended practices may always more self-select themselves into SHG membership and non-response among them are less likely as opposed to their less aware counterparts. These could well generate the potential for selection bias while analyzing the SHG member group and the study samples. Self-selection into SHGs, may result in already existing better practices and behaviors among households with SHG membership confounding the relationship between SHG membership and health outcomes. We tried to minimize these by having a multistage random sampling method to recruit samples with very low non-response. Still the possibility of selection bias should not be ignored. Response bias is also common in studies like serial cross-sectional studies, but given the large universe from where the samples were selected, this was less probable in the current study. In this study the key outcomes were not hard outcomes with definitive clinical endpoints, rather they were mostly behavioral outcomes which have potential for social desirability bias, especially in case of self-reported nature like in this study. Although this kind of information bias are more likely to be non-differential (hence likely to culminate in underestimation of the program effectiveness for most of the binary outcome variables) because in non-JTSP block also there are sources of information regarding recommended practices, still there remains some possibility of such bias. There may also be recall bias given the self-reported nature of study from mothers about their health practices and behaviors, though we minimized the recall period to less than 3 months. There also remains potential for residual bias arising due to comparison of findings over time, although we tried to address this by adjusting for rounds of observation, in our regression models. Separate regression model was also run for each of the outcome variables to avoid issues arising out of multiple comparisons.

By virtue of the large sample size, robust analysis using multiple methods of modeling (Multinomial and Ordinal Multivariable Logistic Regressions) and numerous variables to examine the domains of enquiry, despite the limitations mentioned above, the current study could determine the effectiveness of the JTSP program with considerable precision and validity at the population level—which is a unique strength of the study. Generally, for these kinds of interventions at scale, it is quite impractical and infeasible to determine the attributional path for the population level changes. Hence it is expected that the findings of the current study are likely to inform the system strengthening programmatic efforts for health and nutrition through existing channels in the state of Bihar as well as elsewhere in similar settings.

Finally, the study results suggest successful HN Integration in JEEViKA with a Technical Support program, i.e., JTSP and provide evidence for the effectiveness of JTSP’s multi-pronged social behavior change communication approach of integrating HN within State Rural Livelihoods Mission community platforms, as well as scalability to impact a larger community. JTSP provides learnings on how to work as a technical support program for the rural development and better livelihood working collaboratively within a government system. It provides evidence that the successful HNS integration within the rural livelihoods mission, needs systematic introduction and strengthening at multiple levels. This was achieved through several modalities such as policy-advocacy (at the national level, with partners and with JEEViKA’s leadership), strategy development, capacity strengthening, quality monitoring, all of which enabled JEEViKA systems to own, and self-sustain its HNS agenda and implementation processes. Integration of such technical programs into the system bring forth great promise toward implementation of cross-sectoral comprehensive delivery mechanisms for social development.

Although implementation of behavior change interventions in large, diverse populations is complex and challenging, JTSP has addressed this complexity and employed a systematic approach, focusing on barrier understanding, tailored intervention design, and rigorous implementation. Considering the vast geography of the state of Bihar, it is possible to have variability in outcomes with uneven quality and rigor in interventions across all regions. Moreover, multiple other priorities of Jeevika may have impacted the depth and intensity of HNS-related activities. While JTSP successfully transitioned many roles to JEEViKA’s staff, community leadership in HNS remains underdeveloped. Ensuring that community institutions can truly and sustainably steer the HNS agenda independently is an ongoing challenge that needs more strategic focus and resources. Deeply ingrained cultural norms and socioeconomic barriers in rural Bihar may have also limit the effectiveness of interventions resulting in less optimal participation and addressing these requires long-term engagement and tailored strategies. These programmatic aspects and challenges affecting the outcomes can be addressed in the next phase of the program.

## Data Availability

The datasets generated for this study and used for analysis shall be available on request to the corresponding author.
